# Cause of Death among Long-Term Cancer Survivors: The NANDE Study

**DOI:** 10.3390/healthcare11060835

**Published:** 2023-03-13

**Authors:** Makoto Fujii, Toshitaka Morishima, Mayumi Nagayasu, Haruka Kudo, Yuko Ohno, Tomotaka Sobue, Isao Miyashiro

**Affiliations:** 1 Division of Health Sciences, Graduate School of Medicine, Osaka University, Suita 5650871, Osaka, Japan; 2 Cancer Control Center, Osaka International Cancer Institute Otemae, 3-1-69, Osaka 5418567, Osaka, Japan; 3 Environmental Medicine and Population Sciences, Department of Social Medicine, Graduate School of Medicine, Osaka University, Suita 5650871, Osaka, Japan

**Keywords:** neoplasms, cause of death, registries

## Abstract

Survival information for Japanese patients with cancer is based only on survival status and the cause of death among these patients remains unclear. In this study, Osaka Cancer Registry data (1985–2014) and vital statistics data (1985–2016) were linked to create a database, permitting the extraction of data on the causes of death. In total, 522,566 subjects diagnosed with cancer between 1995 and 2011 were analyzed. Follow-up for vital status was conducted 5 and 10 years after cancer diagnosis. To evaluate the three causes of death (index cancer, non-index cancer, and non-cancer death), cause-specific hazard and cumulative incidence functions were estimated using a life table and Gray’s methods. The number of deaths owing to any of the causes in the observation period (median: 3.51 years, mean: 4.90 years) was 394,146. The 5- and 10-year cancer-specific survival rate was 48.56% and 39.92%, respectively. Immediately after cancer onset, the hazard of index cancer death was high. The proportion of non-index cancer deaths was high in patients with mouth and pharynx cancers. The hazard of index cancer death remained constant for breast and liver cancers. In prostate, breast, and laryngeal cancers with good prognosis, the hazard of non-index cancer and non-cancer death constantly increased.

## 1. Introduction

Cancer accounts for a significant proportion of deaths worldwide, accounting for 5,659,700 deaths in 1990 and over 9 million deaths in 2017, as the number of deaths increases worldwide [1,2]. In Japan, the reported number of deaths from cancer in 2021 was 381,505 (26.5%), and cancer was the leading cause of death. The second leading cause of death in 2021 was heart disease, with 214,710 (14.91%) reported deaths, followed by cerebrovascular disease with 104,595 deaths (7.26%) [3].

Cancer incidence rates continue to increase [4,5,6], although cancer mortality is declining [7,8]. The number of patients with cancer with long-term survival has increased worldwide [9,10]. In addition, with the aging world population, the age of patients with cancer is also increasing. Therefore, it is postulated that more patients will die from causes of death other than primary cancer [5,7]. Few studies have examined the cause of death by cancer site; however, studies examining causes of death limited to breast cancer patients and others that examined survival rates only, without limiting the cause of death by cancer site, have indicated that the incidence of non-cancer death has increased among patients with breast and prostate cancer [11,12]. The Japanese cancer registries only present information on survival status, with information regarding the cause of death remaining unknown. In addition, data such as the proportion of deaths by cause and time trends are lacking. The increase in the number of cancer survivors is a major public health challenge. For cancer survivors to be active in society, it is important to optimize cancer care and provide support and information. Therefore, it is necessary to have an accurate overview of the current situation, including the causes of death among patients with cancer according to cancer type, patient sex and age, and temporal trends in mortality. This study aimed to estimate the hazards of index cancer deaths, non-index cancer deaths, and non-cancer deaths in patients with cancer.

## 2. Materials and Methods

### 2.1. Study Design

This population-based cohort study used a record linkage database of population-based cancer registry data and vital statistical data.

### 2.2. Primary Endpoint

The primary endpoint of this study was the cumulative incidence rate of deaths among patients with cancer, according to cancer site and cause of death.

### 2.3. Creating an Analysis Database

#### 2.3.1. Information on Cancer Incidence

The subjects comprised 1,088,848 patients with cancer, registered in the population-based cancer registry in Osaka prefecture from 1985 to 2014. The Osaka Cancer Registry (OCR) is a well-established registry, based on the complete enumeration of incidence data. It is the largest population-based cancer registry in Japan, with long-term cancer patient registration information. Furthermore, patients were followed up using official resident registries to establish vital status at 5 and 10 years after a cancer diagnosis. A high follow-up rate was maintained (98–99%). For the classification of cancer sites, the third edition of the International Classification of Diseases for oncology topography (ICD-O-3-T) and morphology (ICD-O-3-M) codes were used. In cases where a patient was diagnosed with more than one cancer, we used the information on the first cancer diagnosis.

#### 2.3.2. Information on the Cause of Death

Vital statistics (VS) provide the oldest and most accurate information on the causes of death in Japan. These statistics are based on the death certificates of deceased residents. One cause of death was identified for each patient. Overall, 32,144,355 causes of death were registered in vital nationwide statistics from 1985 to 2016 [3]. Death cause information was classified using the ninth edition of the International Classification of Diseases (ICD-9) code for decedents who died between 1985 and 1994, while the tenth edition of the International Classification of Diseases (ICD-10) code was used after 1995.

#### 2.3.3. Linking Cancer Infection Information and Death Information

The present study was one of the derived studies conducted by the collaborative study group, NANDE (Neoplasms and other Causes of Death), that identifies and characterizes the cause of death in patients with cancer. First, we created a database to examine the causes of death in patients with cancer. No unique identifier (e.g., social security number) was available in the OCR or VS to allow for deterministic linkage of the corresponding records from the same person. Therefore, OCR data and VS were probabilistically linked using nine indicators: prefectures at the time of death, municipalities at the time of death, sex, birth year, birth month, birthday, year of death, month of death, and the date of death. In the OCR, 716,873 patients with cancer were confirmed to have died between 1985 and 2014. Eventually, 692,069 people from the registry data (96.5% of all deceased) were probabilistically linked with the corresponding data from VS using nine indicators (680,261 people) or eight indicators, except for any of the following: birthday, date of death, or municipality at the time of death (11,808 people). Furthermore, 371,918 people, in whom death could not be confirmed ten years after cancer diagnosis, were included. The final database developed for the NANDE study included 1,063,987 patients.

### 2.4. Selection of Participants

To examine the cause of death in patients with cancer, we extracted only cases with accurate information on morbidity and death derived from the full NANDE dataset. Participants diagnosed with cancer from 1995 to 2011 were included in the analysis. In Japan, ICD-10 coding for causes of death has been used since 1995. Therefore, only deaths from 1995 to 2016 were analyzed in this study, because the ICD-9 and ICD-10 codes do not match entirely. The following cases were excluded from the survival analysis: patients diagnosed after 2012 with less than five years of follow-up (165,416 cases, 15.55% of the total), death certificate only (110,471 cases, 10.38% of the total), carcinoma in situ with good prognosis (46,077 cases, 4.33% of the total), patients with a survival time of 0 days, and patients in whom the number of months survived was unknown (133,298 cases, 12.53% of total). Excluding these cases, 522,566 subjects were included in the last analysis from the 1,063,987 patients in the NANDE dataset, corresponding to 49.11% of the total number of cancer cases.

### 2.5. Ethics Approval

In this study, all patient data were de-identified, and the requirement for informed consent requirement was waived. This study was approved by the ethics committee of the Osaka International Cancer Institute (approval number 1707105103).

### 2.6. Cancer Site Classification Code and Cause of Death Classification Code

To classify cancer incidence, the third edition of the International Classification of Diseases was used. Oncology topography (ICD-O-3-T) and morphology (ICD-O-3-M) codes were used. The ICD-10 code was used to classify the causes of death. These categories are lip, oral cavity, and pharynx (ICD-O-3-T or ICD-10: C00-C14), esophagus (C15), stomach (C16), colon and rectum (C18-C20), liver (C22), gallbladder, etc. (C23, C24), pancreas (C25), larynx (C32), trachea, bronchus and lung (C33, C34), bone, joints and articular cartilage (C40, C41, C47, C49), skin (C44), breast (C50), cervix uteri (C53), corpus uteri (C54), ovary (C56), prostate (C61), kidney and urinary tract (C62-C66, C68), bladder (C67), thyroid (C73), malignant lymphomas and Hodgkin lymphomas (ICD-O-3-M: 959–972, 974–975, ICD-10: C817-C859), multiple myeloma (ICD-O-3-M: 973, 976, ICD-10: C900), and leukemia (ICD-O-3-M: 980–994, ICD-10: C910-C959). In this study, using the above codes, the index cancer death (cancer death at the same site as the incident primary cancer), non-index cancer death (cancer death at a site different from the incident primary cancer), and non-cancer-related causes of death were examined (Table 1). Detailed causes of death were classified according to the classification table of causes of death provided by the Japanese Ministry of Health, Labour and Welfare (Table A1).

### 2.7. Statistical Analysis

The 5- and 10-year crude observed survival rate was calculated using the Kaplan–Meier method [13], and corrected survival rates were calculated by treating the other cause of deaths occurring with five or ten years of follow-up as withdrawals. For example, for calculating the adjusted survival rate for index cancer deaths, non-index cancer deaths, and non-cancer deaths were considered censored. The corrected survival rate of 50% for index cancer death indicates that 50% of index cancer patients are spared the risk of death from the disease within 10 years of diagnosis [14]. The proportion of deaths at the last follow-up was calculated for each cancer site and sex. We created a Sankey diagram to show the correlation between the site of cancer incidence and the ultimate cause of death in relation to gender. Hazard rates were calculated for the following three causes of death using the life table method every half year: index cancer death, non-index cancer death, and non-cancer death [15]. In addition, the hazard ratio was calculated using the cancer-specific hazard rate as the denominator, and the non-index cancer-specific non-cancer-specific hazard rate as the numerator. To estimate the cumulative hazard function by competing for the cause of death, we used the Fine and Gray method, with a proportional hazards model for the sub-distribution of a competing risk [16,17]. The significance level was 5% on both sides, and all statistical analyses were performed using SAS (version 9.4; SAS Institute, Cary, NC, USA).

## 3. Results

A total of 327,954 patients died by any cause of death in the observation period (median: 3.51 years, mean: 4.90 years). The proportion of index cancer deaths was 51.83% for male patients and 42.96% for female patients, while the proportion of non-index cancer deaths was 7.88% for male patients and 6.35% for female patients, and the proportion of non-cancer deaths was 8.58% for male patients and 5.91% for female patients. The 5- and 10-year cancer-specific survival rate was 48.56% (48.42–48.70%) and 39.92% (39.77–40.07%), respectively.

Table 2 and Table 3 summarize the 10-year crude and corrected survival rates by the cause of death and cancer site. When all sites were considered together, female patients had higher crude survival rates than male patients (male patients: 31.55% vs. female patients: 54.37%; Table 2 and Table 3). This tendency was evident for mouth and pharynx cancers (male patients: 35.02% vs. female patients: 48.45%), esophageal cancer (16.58% vs. 24.49%), and lung cancer (11.42% vs. 20.29%). In contrast, female patients had lower crude survival rates than male patients for bladder cancer (male patients: 46.95% vs. female patients: 43.62%).

In male patients, the cancer with the highest 10-year observed survival rate was thyroid cancer (67.99%, 95% CI: 65.63–70.22%), followed by breast cancer (58.14%, 95% CI: 51.54–64.17%), laryngeal cancer (52.69%, 95% CI: 51.13–54.24%), prostate cancer (52.58%, 95% CI: 51.81–53.35%), and bladder cancer (46.95%, 95% CI: 45.98–47.91%). In contrast, the lowest 10-year survival rate was for pancreatic cancer (3.78%, 95% CI: 3.39–4.20%), followed by liver cancer (8.33%, 95% CI: 8.02–8.65%), gall bladder cancer (10.39%, 95% CI: 9.60–11.21%), lung cancer (11.42%, 95% CI: 11.12–11.72%), and multiple myeloma (11.43%, 95% CI: 9.78–13.21%). In female patients, the cancer with the highest 10-year survival rate was thyroid cancer (80.58%, 95% CI: 79.50–81.62%), followed by breast cancer (71.47%, 95% CI: 71.06–71.88%), corpus uteri cancer (68.61%, 95% CI: 67.51–69.69%), cervix uteri cancer (64.95%, 95% CI: 64.04–65.83%), and laryngeal cancer (55.84%). Conversely, the lowest 10-year observed survival rate was for pancreatic cancer (4.37%, 95% CI: 3.91–4.85%), followed by liver cancer (8.56%, 95% CI: 8.06–9.08%), gall bladder cancer (10.97%, 95% CI: 10.23–11.5%), multiple myeloma (14.85%, 95% CI: 12.93–16.90%), and lung cancer (20.29%, 95% CI: 19.71–20.88%).

In male patients, the cancer with the highest 10-year cause-specific survival rate was skin cancer (87.13%, 95% CI: 85.78–88.35%), followed by thyroid cancer (81.52%, 95% CI: 79.54–83.34%), and laryngeal cancer (80.31%, 95% CI: 79.03–81.53%). In contrast, the lowest 10-year cause-specific survival rate was for pancreatic cancer (5.85%, 95% CI: 5.36–6.37%), followed by liver cancer (13.09%, 95% CI: 12.65–13.54%), gall bladder cancer (17.00%, 95% CI: 15.97–18.05%), lung cancer (17.07%, 95% CI: 16.71–17.44%), and multiple myeloma (19.48%, 95% CI: 17.06–22.03%). In female patients, the cancer with the highest 10-year cause-specific survival rate was skin cancer (88.75%, 95% CI: 87.43–89.93%), followed by thyroid cancer (88.56%, 95% CI: 87.70–89.36%), corpus uteri cancer (80.87%, 95% CI: 79.94–81.76%), laryngeal cancer (80.16%, 95% CI: 75.26–84.18%), and breast cancer (80.16%, 95% CI: 75.26–84.18%). Conversely, the lowest 10-year cause-specific survival rate was for pancreatic cancer (5.89%, 95% CI: 5.35–6.46%), followed by liver cancer (13.58%, 95% CI: 12.88–14.30%), gall bladder cancer (16.10%, 95% CI: 15.20–17.02%), multiple myeloma (21.00%, 95% CI: 18.54–23.57%), and lung cancer (25.21%, 95% CI: 24.57–25.85%).

Table 4 and Table 5 summarize the 5-year crude and corrected survival rates by the cause of death and cancer site. These survival rates are estimated at 5 years after cancer diagnosis and show a similar trend to the 10-year survival rates, with all crude observed survival rates being higher at 5 years. In corrected survival rates, there is a difference of only a few percentage points in index cancer death between the 5-year and 10-year estimates. However, there is a nearly 10% difference in non-index cancer death and non-cancer death estimates.

**Figure A1:**
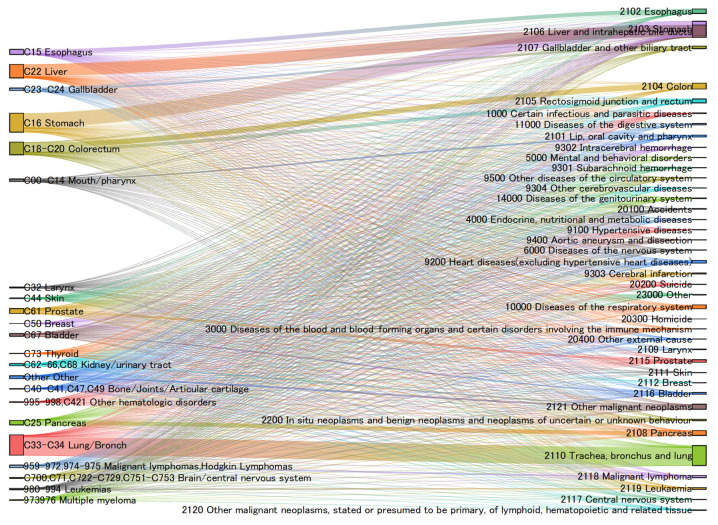
Relationships between cancer sites and cause of death (male patients).

**Figure A2:**
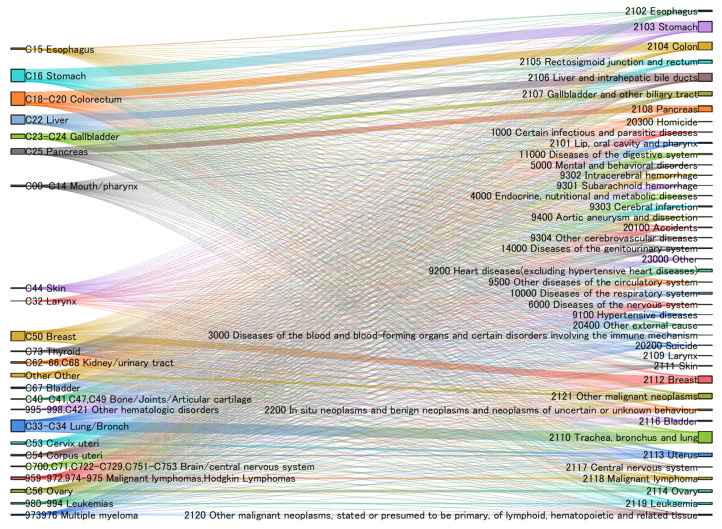
Relationships between cancer sites and cause of death (female patients).

Pancreatic cancer had the highest proportion of index cancer deaths in male patients (pancreatic cancer death, 89.21%; other cancer death, 2.81%; and non-cancer death, 2.83%). The next highest was lung cancer (lung cancer death, 76.26%; other cancer death, 3.46%; and non-cancer death, 5.58%). In female patients, the cancer with the highest proportion of index cancer deaths was the pancreas (pancreatic cancer death, 89.30%; other cancer death, 2.59%; and non-cancer death, 2.28%). The next highest was the gall bladder (gall bladder cancer death 78.34%, other cancer death 4.71%, and non-cancer death 3.98%). Patients with a poor prognosis had a low proportion of other cancers and non-cancers. Our results revealed that index cancer deaths of the pancreas, lung, gall bladder, liver, esophagus, multiple myeloma, leukemia, and malignant lymphoma were high in male patients, and the index cancer deaths of the pancreas, gall bladder, liver, lung, multiple myeloma, esophagus, leukemia, ovary, and malignant lymphoma were high in female patients. In male patients with mouth and pharynx cancer, the proportion of non-cancer death was 11.47%, which was higher than that of other cancers. This tendency was not observed in female patients. Figure A1 and Figure A2 summarize the relationship between the site of cancer incidence and the ultimate cause of death in a diagram. The composition of causes of death shows that deaths due to cancer at the incidence site are common, and among causes other than cancer, deaths due to cardiovascular disease and respiratory disease are common.

**Figure 1:**
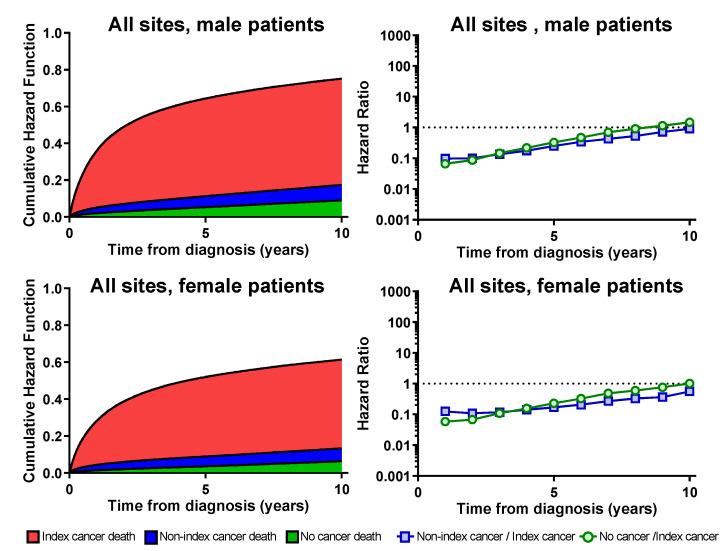
Cumulative hazard rate and hazard ratio by cause of death (all sites). The red area shows the index cancer death, the blue area shows the other cancer deaths, the green area shows the non-cancer deaths, and the other white area shows the cumulative survival rate.

**Figure 2:**
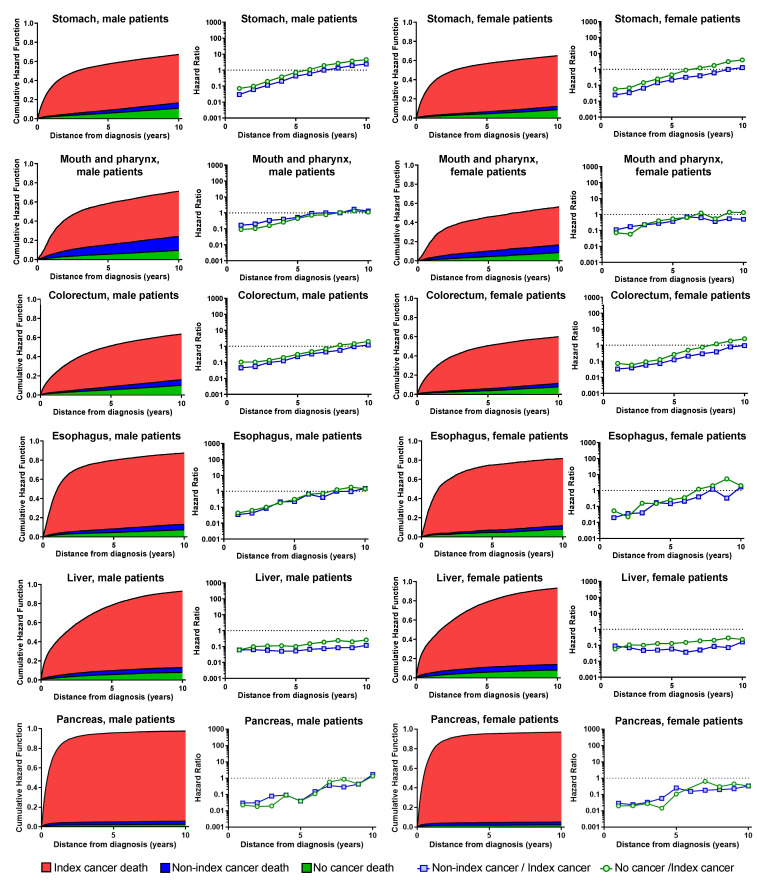

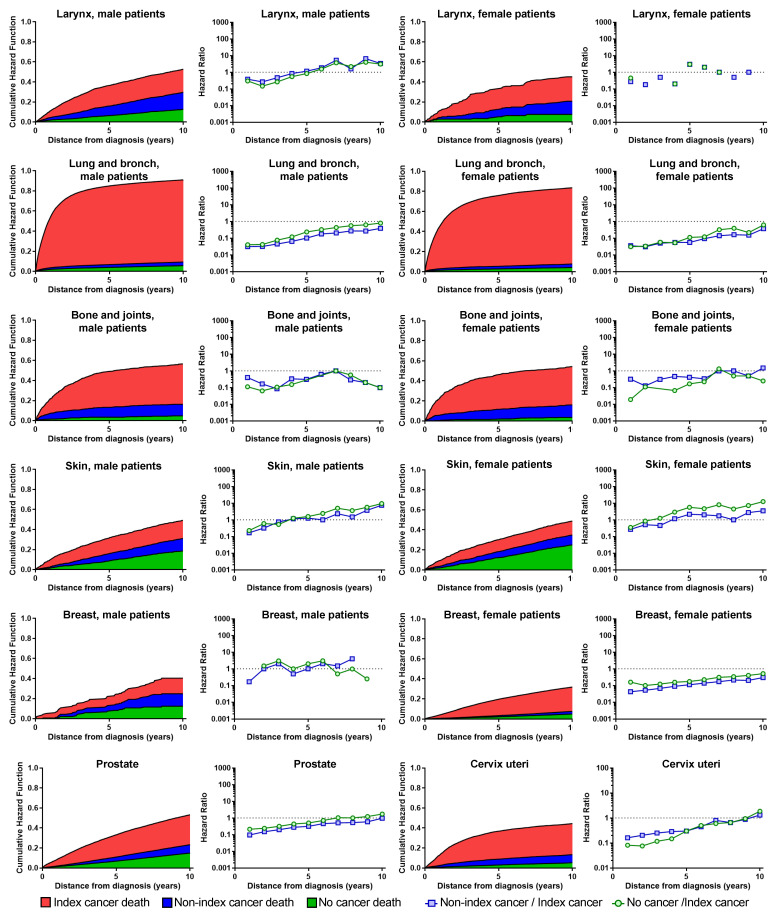

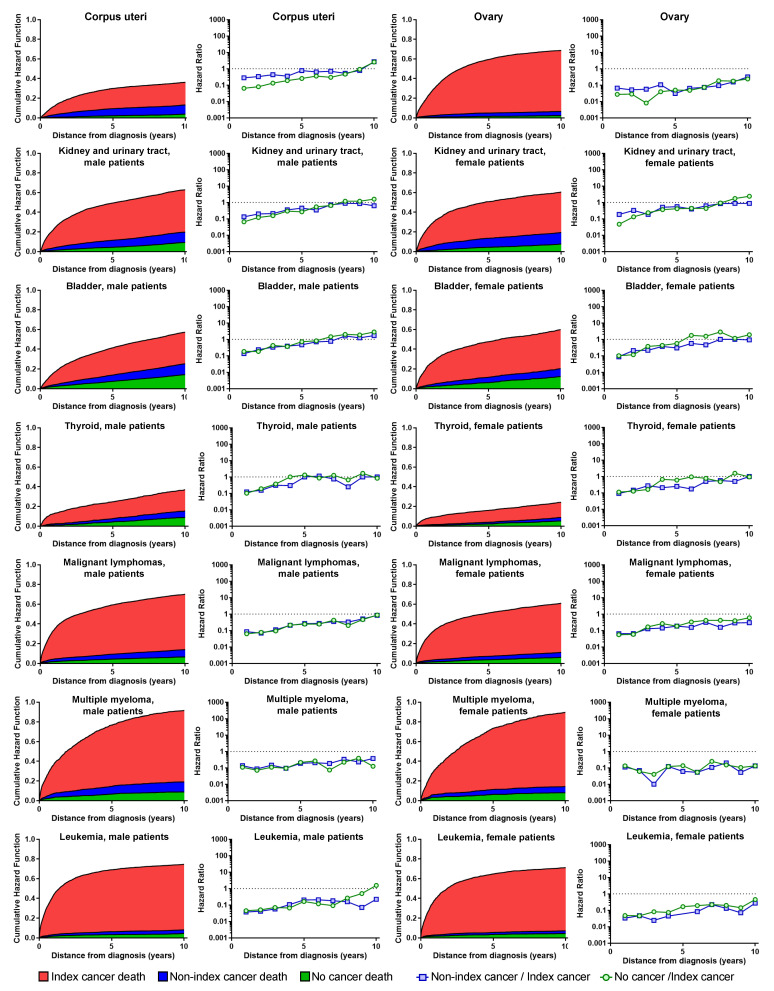
Cumulative hazard rate and hazard ratio by cause of death. The red area shows the index cancer death, the blue area shows the other cancer deaths, the green area shows the non-cancer deaths, and the other white area shows the cumulative survival rate.

Figure 1 and Figure 2 show the temporal trend of the cumulative hazard function of the three causes and the hazard ratio for 6 months. The cumulative hazard function curve shows the cumulative proportion of the causes of death. The red area shows the index cancer death, the blue area shows the other cancer deaths, the green area shows the non-cancer deaths, and the other white area shows the cumulative survival rate.

This tendency was different for each cancer site, and four characteristic types were found. In the first type, the hazard rate of the index cancer was not stable and the index cancer mortality was observed over a long period. This type includes liver cancer, breast cancer in female patients, ovarian cancer, multiple myeloma, and leukemia. With respect to these sites, the hazard of index cancer death does not decrease with secular change. Even after ten years, there is a significant difference between the index cancer death, and non-index cancer deaths and non-cancer deaths. In the second type, most patients die from the index cancer immediately after the onset of cancer. This type included pancreatic, gallbladder, and lung cancer, with over 75% of patients dying from the index cancer. In the third type, including laryngeal cancer in female patients, and skin cancer, and breast cancer in male patients, with relatively low cancer incidence, the confidence intervals of the three causes of death overlap immediately after the onset of cancer. In the fourth type, including prostate, skin, and laryngeal cancers in male patients, index cancer mortality tends to decrease over time.

The hazard ratio shows whether non-index or non-cancer mortality is higher than index cancer mortality. In the first and second types, hazard ratios of both non-index cancer death and no-cancer death were always below 1. In the other cancer sites, the hazard rate of death from the index cancer increased immediately after the onset of cancer; the hazard rate became constant for approximately five years and was almost the same as that of other cancer deaths and non-cancer deaths in 8 to 10 years.

## 4. Discussion

This study clarified the long-term characteristics of the cause of death other than cancer in Japanese patients with cancer. While few studies have examined the cause of death by cancer site, previous studies that were limited to breast cancer patients or those that examined survival rates only, without limiting the cause of death by cancer site, have reported results that were similar to those of this study [11,12,18,19,20,21,22]. In Japan, there is scarce research on the cause of death among patients with cancer and, to the best of our knowledge, the present study is the first large-scale study to examine the characteristics of the cause of death of patients with cancer in Japan. In addition, this research is novel because it can characterize cancer deaths among patients with cancer by linking the OCR, which is the total registration of cancer incidence, and VS, which represent a complete registry of causes of death in the Japanese population. By dint of its population-based cancer registry, Japan was the only country in Asia that could provide data in the CONCORD study, which estimated the relative survival rate of cancer worldwide [9,23]. The OCR was one of the three prefectures participating in the CONCORD study, and this registry is highly regarded worldwide for its scale and accuracy.

### 4.1. Cancer Sites with Consistently High Index Cancer Deaths over the Long Term

There are few studies on the hazards by cause of death for all cancer sites in the 10-year follow-up period after cancer incidence. To the best of our knowledge, few studies have performed an analysis that considers competing risks. These four characteristic types of trends are consistent with those of previous studies. Mortality of patients with breast and liver cancer remains high more than 5 years after cancer, similar to previous studies [12]. In this study, similar trends were observed for liver, ovarian, multiple myeloma, and leukemia; however, there are no previous studies on sites other than breast cancer [11]. In breast cancer, hazards for non-index cancer deaths and non-cancer deaths showed a constant increase in the decade after diagnosis, although this was not observed for liver cancer, ovarian cancer, multiple myeloma, and leukemia. The difference in prognosis between breast cancer and cancer at other sites may explain these results.

In pancreatic, gallbladder, and lung cancer, which are generally considered to have a poor prognosis, the hazard of index cancer death is particularly high, and the hazard declines depending on age after diagnosis. However, it was higher than that of non-index cancer and non-cancer deaths. The 10-year relative survival rate for these cancer sites is very low [8]. In this study, the death proportion of index cancer deaths was 75% or above within 5 years of a cancer diagnosis. Because lung or liver cancers are sites that are likely to be an organ to which primary cancer metastasizes from other organs, if the primary cancer is not listed in the death certificate, it may become the cause of death.

However, if a death certificate states multiple cancer sites, other cancers may be selected as the cause of death, in accordance with the original death cause selection rule. For this reason, the hazard of death from index lung and liver cancer in this study may have been underestimated [24]. It is not possible to directly confirm the contents of the death certificate. However, we consider that this study uses the most robust data currently available.

### 4.2. Cancer Sites with Consistently High Rates of Both Index Cancer Deaths and Non-Index Cancer Deaths

Among male patients, cancer with high mortality from original cancer and high mortality from other cancers are mouth cancer and pharynx cancer, which is different from the trends for the other cancers studied herein. Mouth and pharynx cancers are affected by field cancerization, a phenomenon widely spread across multiple areas due to long-term exposure to common cancer-inducing factors. In previous studies, mouth and pharynx cancers were often affected by esophageal cancer and secondary lung cancer [25]. In this study, male patients with mouth and pharynx cancer had a high risk of death from other cancers, mainly esophageal cancer (15.35%), followed by lung cancer (14.89%); these results are consistent with existing literature.

### 4.3. Cancer Sites with a Relative Decrease in Index Cancer Deaths over the Long Term

Prostate cancer, laryngeal cancer, and breast cancer in female patients showed constant increases in the hazards of other cancer deaths and non-cancer deaths when evaluating the elapsed years after morbidity. Previous prostate and breast cancer studies have shown that the proportion of non-cancer-related our results findings of the present study [11,26,27,28]. In this study, similar trends are also seen in laryngeal cancer, although there is no previously reported research on this point. These three cancer sites are known to have relatively good prognoses. However, similar trends were not observed in thyroid cancer, which is considered to have a good prognosis. In prostate cancer, the age at diagnosis was the highest, and a high frequency of deaths within 5 to 10 years was observed. Although the number of laryngeal cancers was small, similar trends were also observed. Conversely, as patients with thyroid cancer were diagnosed at a young age, it was considered that other cancer deaths and non-cancer deaths cannot be observed in the observation period of 10 years. In this study, prostate cancer had the highest risk of other cancer and noncancer mortality. Previous studies have found that men diagnosed with prostate cancer are less likely to die from prostate cancer, which is consistent with the results of high other cancer and noncancer deaths [26]. However, deaths due to prostate cancer in this study were not consistent with those reported by previous studies because the mortality rate was constant for 10 years after cancer diagnosis [26]. Potential reasons for this discrepancy include the characteristics of the Japanese medical insurance system, wherein even nonfatal cancers are treated aggressively, and the characteristics that make cancer more likely to be selected as the underlying disease if the cause of death selection rules are followed; however, it is difficult to examine this issue in detail from the present data [24]. In prostate cancer, the age at diagnosis is typically high, and it is necessary to further understand the incidence of second cancers during the observation period of 10 years after treatment. In Japan, a national health insurance system is in place, and even elderly patients attending hospitals and diagnosed with cancer are actively treated. In other countries, aggressive treatment for elderly patients with cancer is not undertaken. According to our data on cancer care in the Japanese medical insurance system, all people of all ages are equally admitted to a hospital if diagnosed with cancer, and have the opportunity to receive treatment; selection bias in treatment is considered to be strong.

This study has several limitations. First, it is difficult to analyze patient characteristics and treatment because the registry and vital statistics data do not include clinical information. In addition, there is no adjustment for age at diagnosis and classification by year of birth (for cohort effects). Second, the detailed contents of the death certificate could not be obtained. The wording of the death certificate is primarily influenced by the discretion of the doctor who conducted the death diagnosis, so we could not verify whether the cause of death on the death obtained was the correct cause of death for each patient.

We believe that this study will help people affected by cancer to learn about complications and other diseases that they should be aware of and to review their lifestyle with medical care providers and other stakeholders. The strength of this research is that it is the first study in Japan simultaneously investigating information on cancer incidence and information on the cause of death among patients with cancer. We focused on three causes of death without limiting the cancer sites. In the future, we believe that our study methods will lead to analysis according to personal characteristics such as age at cancer diagnosis, aging, treatment method, degree of clinical progress, and multiple cancer morbidity. In addition, we believe this indicates the need for a study to distinguish the causes of death in more detail and to evaluate the relationship between affected cancer sites and cause of death.

## 5. Conclusions

This study clarified secular trends in the three causes of death after cancer diagnosis and the characteristics of each cancer site. These results indicate that many fatalities caused by index cancers occur immediately after cancer incidence, hazards decrease with time, and the difference between non-index cancer deaths and non-cancer deaths disappears in approximately five to eight years. The results also suggest that in cases of breast and liver cancer with many recurrences, the hazard of the original cancer death does not decrease within 10 years. Furthermore, in prostate, breast, and laryngeal cancers with a relatively good prognosis, non-index cancer deaths and non-cancer deaths increase. These findings are useful for developing strategies against cancer and providing important information to patients. We consider that these data will contribute to the optimization of cancer care and support the provision of appropriate information for cancer survivors.

## Figures and Tables

**Table 1:** Correspondence between ICD-O-3 T and ICD-O-3 M codes on cancer incidence, and IDC-10 code on the cause of death.

Primary Cancer Site (ICD-O-3 T or ICD-O-3 M)	Cause of Death (ICD-10)
Mouth, pharynx (C00–C14)	Mouth, pharynx (C00–C14)
Esophagus (C15)	Esophagus (C15)
Stomach (C16)	Stomach (C16)
Colorectum (C18–C20)	Colorectum (C18–C20)
Liver (C22)	Liver (C22)
Gallbladder (C23–C24)	Gallbladder (C23–C24)
Pancreas (C25)	Pancreas (C25)
Larynx (C32)	Larynx (C32)
Lung, bronchial (C33–C34)	Lung, bronchial (C33–C34)
Bone, joints, articular cartilage (C40–C41, C47, C49)	Bone, joints, articular cartilage (C40–C41, C47, C49)
Skin (C44)	Melanoma, skin (C43, C44)
Breast (C50)	Breast (C50)
Cervix uteri (C53)	Cervix uteri (C539)
Corpus uteri (C54)	Corpus uteri (C549)
Ovary (C56)	Ovary (C56)
Prostate (C61)	Prostate (C61)
Kidney, urinary tract (C62–C66, C68)	Kidney, urinary tract (C62–C66, C68)
Bladder (C67)	Bladder (C67)
Thyroid (C73)	Thyroid (C73)
Malignant lymphomas, Hodgkin lymphomas (959–972, 974–975)	Malignant lymphomas, Hodgkin lymphomas (C817–C859)
Multiple myeloma (973, 976)	Multiple myeloma (C900)
Leukemias (980–994)	Leukemias (C910–C959)

ICD-O-3-T denotes the third edition of the International Classification of Diseases for oncology topography, ICD-O-3-M the third edition of the International Classification of Diseases for oncology morphology, and ICD10 the International Statistical Classification of Diseases and Related Health Problems, 10th Revision.

**Table 2:** Summary of cancer morbidity and 10-year cancer mortality by cause of death (male, incidence: 1995–2011, death or survival: 1995–2016).

Site	Male									
		All Cause		Index Cancer Death		Non-Index Cancer Death		Non-Cancer Death		Censor
	Incidence	Death	10-Year Observed Survival Rate	Death	10-Year Corrected Survival Rate	Death	10-Year Corrected Survival Rate	Death	10-Year Corrected Survival Rate	
	*n*	*n*	% (95% CI)	*n*	% (95% CI)	*n*	% (95% CI)	*n*	% (95% CI)	*n*
All	301,707	205,999	31.55 (31.38–31.72)	156,360	–	23,764	–	25,875	–	95,708
Mouth, pharynx	7964	5163	35.02 (33.98–36.06)	3468	54.46 (53.34–55.56)	997	77.85 (76.56–79.07)	698	82.66 (81.44–83.82)	2801
Esophagus	12,456	10,248	16.58 (15.89–17.29)	8639	27.34 (26.51–28.18)	702	80.61 (79.04–82.08)	907	75.39 (73.71–76.99)	2208
Stomach	61,403	38,097	40.26 (39.89–40.63)	28,511	56.32 (55.96–56.69)	3281	89.19 (88.84–89.52)	6305	80.17 (79.75–80.59)	23,306
Colorectum	44,981	25,554	42.92 (42.47–43.37)	19,056	57.46 (57.02–57.91)	2361	89.71 (89.31–90.09)	4137	83.28 (82.82–83.74)	19,427
Liver	31,680	28,254	8.33 (8.02–8.65)	23,854	13.09 (12.65–13.54)	1654	86.39 (85.53–87.19)	2746	73.86 (72.78–74.92)	3426
Gallbladder	6015	5398	10.39 (9.60–11.21)	4681	17.00 (15.97–18.05)	410	78.69 (76.17–80.99)	307	78.00 (75.09–80.61)	617
Pancreas	10,087	9706	3.78 (3.39–4.2)	9150	5.85 (5.36–6.37)	284	83.65 (80.17–86.57)	272	77.82 (73.80–81.30)	381
Larynx	3506	1631	52.69 (51.13–54.24)	695	80.31 (79.03–81.53)	534	79.09 (77.57–80.51)	402	82.97 (81.53–84.32)	1875
Lung/bronchial	48,217	42,451	11.42 (11.12–11.72)	38,010	17.07 (16.71–17.44)	1721	86.62 (85.85–87.35)	2720	77.37 (76.43–78.27)	5766
Bone, joints, articular cartilage	1258	666	50.81 (48.26–53.30)	478	62.50 (59.91–64.98)	124	87.30 (85.17–89.14)	64	93.13 (91.31–94.58)	592
Skin	2875	1183	54.18 (52.21–56.12)	348	87.13 (85.78–88.35)	321	83.42 (81.65–85.04)	514	74.58 (72.59–76.45)	1692
Breast	225	90	58.14 (51.54–64.17)	37	80.19 (74.04–85.03)	21	85.79 (79.51–90.26)	32	84.46 (78.76–88.75)	135
Prostate	26,010	9656	52.58 (51.81–53.35)	4979	74.93 (74.24–75.60)	1743	87.75 (87.12–88.35)	2934	80.05 (79.31–80.76)	16,354
Kidney, urinary tract	7729	4222	42.83 (41.70–43.96)	2895	60.07 (58.93–61.18)	681	85.35 (84.27–86.37)	646	83.60 (82.39–84.73)	3507
Bladder	9615	5035	46.95 (45.98–47.91)	2813	70.13 (69.23–71.01)	969	84.50 (83.61–85.35)	1253	79.27 (78.28–80.23)	4580
Brain, central nervous system	2030	1554	27.31 (25.51–29.14)	798	50.78 (48.35–53.15)	579	63.84 (61.33–66.23)	177	84.44 (82.05–86.53)	476
Thyroid	1629	483	67.99 (65.63–70.22)	283	81.52 (79.54–83.34)	85	92.69 (91.09–94.02)	115	89.94 (88.13–91.49)	1146
Malignant lymphomas, Hodgkin lymphomas	7869	4877	37.02 (35.93–38.11)	3850	48.84 (47.69–49.98)	521	87.17 (86.03–88.22)	506	87.04 (85.90–88.09)	2992
Multiple myeloma	1634	1396	11.43 (9.78–13.21)	1102	19.48 (17.06–22.03)	154	77.40 (72.93–81.23)	140	76.06 (71.38–80.08)	238
Leukemias	4360	3058	30.49 (29.16–31.83)	2712	36.27 (34.86–37.69)	171	91.92 (90.60–93.06)	175	91.51 (90.08–92.75)	1302

Excluded other cancer *n* = 10,164, 95% CI denotes 95% confidence intervals. All survival rates in the table are crude survival rates.

**Table 3:** Summary of cancer morbidity and 10-year cancer mortality by cause of death (female, incidence: 1995–2011, death or survival: 1995–2016).

Site	Female									
		All Cause		Index Cancer Death		Non-Index Cancer Death		Non-Cancer Death		Censor
	Incidence	Death	10-Year Observed Survival Rate	Death	10-Year Corrected Survival Rate	Death	10-Year Corrected Survival Rate	Death	10-Year Corrected Survival Rate	
	*n*	*n*	% (95% CI)	*n*	% (95% CI)	*n*	% (95% CI)	*n*	% (95% CI)	*n*
All	220,859	121,955	45.63 (45.43–45.83)	94,887	–	14,020	–	13,048	–	98,904
Mouth, pharynx	3336	1717	48.45 (46.81–50.07)	1213	62.68 (61.06–64.26)	239	88.98 (87.62–90.19)	265	86.93 (85.42–88.29)	1619
Esophagus	2517	1870	24.49 (22.74–26.28)	1588	35.75 (33.86–37.64)	91	89.74 (87.19–91.81)	191	76.42 (73.02–79.45)	647
Stomach	28,526	17,025	43.60 (43.06–44.14)	13,819	55.16 (54.63–55.69)	908	93.82 (93.43–94.19)	2298	84.27 (83.69–84.82)	11,501
Colorectum	33,782	18,063	46.64 (46.12–47.16)	14,614	57.52 (57.01–58.02)	1051	93.82 (93.46–94.17)	2398	86.45 (85.95–86.94)	15,719
Liver	13,923	12,315	8.56 (8.06–9.08)	10,254	13.58 (12.88–14.3)	816	86.26 (84.90–87.50)	1245	73.36 (71.66–74.98)	1608
Gallbladder	6531	5896	10.97 (10.23–11.75)	5320	16.10 (15.2–17.02)	313	83.05 (80.69–85.16)	263	82.35 (79.76–84.63)	635
Pancreas	8372	8027	4.37 (3.91–4.85)	7613	5.89 (5.35–6.46)	224	88.20 (85.34–90.54)	190	84.46 (80.58–87.63)	345
Larynx	269	109	55.84 (50.13–61.15)	56	80.16 (75.26–84.18)	30	82.31 (76.78–86.64)	23	84.63 (79.16–88.77)	160
Lung/bronchial	20,470	16,004	20.29 (19.71–20.88)	14,568	25.21 (24.57–25.85)	671	91.65 (90.86–92.37)	765	87.91 (86.93–88.82)	4466
Bone, joints, articular cartilage	992	476	54.98 (52.09–57.76)	327	67.47 (64.66–70.12)	115	86.08 (83.57–88.23)	34	94.66 (92.76–96.07)	516
Skin	2781	1080	55.8 (53.73–57.82)	288	88.75 (87.43–89.93)	220	88.65 (87.05–90.06)	572	70.93 (68.80–72.95)	1701
Breast	45,842	11,765	71.47 (71.06–71.88)	8682	78.81 (78.43–79.18)	1057	96.66 (96.46–96.84)	2026	93.85 (93.60–94.09)	34,077
Cervix uteri	8292	3244	64.95 (64.04–65.83)	2336	75.99 (75.18–76.77)	545	91.24 (90.60–91.84)	363	93.67 (93.08–94.21)	5048
Corpus uteri	6577	2038	68.61 (67.51–69.69)	1259	80.87 (79.94–81.76)	577	89.26 (88.43–90.04)	202	95.05 (94.38–95.65)	4539
Ovary	6721	4153	39.34 (38.20–40.48)	3736	44.26 (43.08–45.42)	279	92.97 (92.10–93.75)	138	95.66 (94.88–96.33)	2568
Kidney, urinary tract	3574	1927	44.89 (43.24–46.53)	1310	61.17 (59.54–62.76)	346	85.27 (83.73–86.68)	271	86.12 (84.45–87.63)	1647
Bladder	2819	1578	43.62 (41.85–45.37)	1056	61.83 (60.08–63.53)	220	87.05 (85.40–88.52)	302	81.05 (79.11–82.83)	1241
Brain, central nervous system	1700	1285	28.52 (26.52–30.54)	606	54.87 (52.23–57.42)	536	61.07 (58.30–63.71)	143	85.25 (82.70–87.45)	415
Thyroid	5071	930	80.58 (79.50–81.62)	574	88.56 (87.70–89.36)	140	96.34 (95.74–96.86)	216	94.44 (93.72–95.08)	4141
Malignant lymphomas, Hodgkin lymphomas	6259	3326	45.4 (44.14–46.66)	2690	55.32 (54.05–56.56)	279	92.25 (91.27–93.13)	357	89.03 (87.88–90.07)	2933
Multiple myeloma	1495	1220	14.85 (12.93–16.90)	1017	21 (18.54–23.57)	94	84.62 (80.65–87.84)	109	83.98 (80.30–87.03)	275
Leukemias	3215	2164	33.59 (32.04–35.15)	1958	38.2 (36.58–39.82)	83	95.16 (93.97–96.13)	123	92.46 (90.99–93.70)	1051

Excluded other cancer *n* = 7795, 95% CI denotes 95% confidence intervals. All survival rates in the table are crude survival rates.

**Table 4:** Summary of cancer morbidity and 5-year cancer mortality by cause of death (male, incidence: 1995–2011, death or survival: 1995–2016).

Site	Male									
		All Cause		Index Cancer Death		Non-Index Cancer Death		Non-Cancer Death		Censor
	Incidence	Death	5-Year Crude Observed Survival Rate	Death	5-Year Corrected Survival Rate	Death	5-Year Corrected Survival Rate	Death	5-Year Corrected Survival Rate	
	*n*	*n*	% (95% CI)	*n*	% (95% CI)	*n*	% (95% CI)	*n*	% (95% CI)	*n*
All	301,707	181,607	39.82 (39.64–39.99)	145,938	–	18,155	–	17,514	–	120,100
Mouth, pharynx	7964	4399	44.72 (43.63–45.81)	3216	56.46 (55.31–57.60)	711	87.07 (86.12–87.95)	472	91.01 (90.18–91.77)	3565
Esophagus	12,456	9497	23.75 (23.01–24.50)	8374	29.38 (28.55–30.22)	484	91.23 (90.39–92.01)	639	88.67 (87.73–89.54)	2959
Stomach	61,403	33,384	45.63 (45.24–46.03)	27,418	53.60 (53.20–54.01)	1988	94.70 (94.46–94.93)	3978	89.92 (89.61–90.22)	28,019
Colorectum	44,981	21,430	52.39 (51.93–52.85)	17,336	59.77 (59.30–60.23)	1435	95.40 (95.15–95.63)	2659	91.90 (91.60–92.20)	23,551
Liver	31,680	24,300	23.35 (22.88–23.82)	20,786	29.13 (28.60–29.67)	1390	92.38 (91.94–92.78)	2124	86.81 (86.23–87.36)	7380
Gallbladder	6015	5177	13.87 (13.01–14.76)	4562	18.39 (17.35–19.46)	366	85.42 (83.71–86.96)	249	88.39 (86.67–89.90)	838
Pancreas	10,087	9559	5.13 (4.71–5.57)	9058	6.22 (5.74–6.74)	261	90.97 (89.29–92.39)	240	90.73 (88.92–92.26)	528
Larynx	3506	1225	65.04 (63.44–66.60)	633	80.51 (79.09–81.84)	341	88.43 (87.21–89.54)	251	91.38 (90.29–92.35)	2281
Lung/bronchial	48,217	40,118	16.76 (16.43–17.10)	36,659	20.26 (19.89–20.64)	1383	93.16 (92.74–93.55)	2076	88.86 (88.33–89.37)	8099
Bone, joints, articular cartilage	1258	594	52.73 (49.92–55.44)	434	62.64 (59.76–65.37)	109	89.10 (86.95–90.91)	51	94.50 (92.79–95.81)	664
Skin	2875	854	70.28 (68.57–71.92)	312	88.47 (87.20–89.63)	214	91.32 (90.14–92.37)	328	86.99 (85.61–88.25)	2021
Breast	225	60	73.33 (67.04–78.62)	26	87.67 (82.40–91.44)	11	94.45 (90.19–96.90)	23	88.56 (83.27–92.25)	165
Prostate	26,010	6684	74.33 (73.79–74.85)	3932	84.20 (83.74–84.65)	1012	95.49 (95.21–95.76)	1740	92.44 (92.09–92.78)	19,326
Kidney, urinary tract	7729	3517	54.48 (53.37–55.59)	2624	64.28 (63.16–65.37)	499	91.35 (90.58–92.05)	394	92.81 (92.09–93.48)	4212
Bladder	9615	3928	59.13 (58.14–60.10)	2515	72.09 (71.15–73.01)	632	91.52 (90.86–92.13)	781	89.63 (88.91–90.31)	5687
Brain, central nervous system	2030	1441	28.92 (26.95–30.91)	745	52.00 (49.38–54.55)	545	63.44 (60.78–65.97)	151	87.77 (85.62–89.62)	589
Thyroid	1629	355	78.18 (76.09–80.11)	237	85.17 (83.32–86.82)	48	96.59 (95.5–097.42)	70	95.04 (93.77–96.05)	1274
Malignant lymphomas, Hodgkin lymphomas	7869	4241	46.10 (44.99–47.19)	3507	53.36 (52.21–54.49)	375	92.95 (92.21–93.63)	359	92.96 (92.20–93.65)	3628
Multiple myeloma	1634	1211	25.80 (23.69–27.95)	973	34.25 (31.76–36.74)	124	86.29 (83.64–88.53)	114	87.37 (84.80–89.53)	423
Leukemias	4360	2871	34.14 (32.73–35.56)	2590	38.40 (36.91–39.89)	140	94.33 (93.28–95.22)	141	94.29 (93.24–95.19)	1489

Excluded other cancer *n* = 10,164, 95% CI denotes 95% confidence intervals. All survival rates in the table are crude survival rates.

**Table 5:** Summary of cancer morbidity and 5-year cancer mortality by cause of death (female, incidence: 1995–2011, death or survival: 1995–2016).

Site	Female									
		All Cause		Index Cancer Death		Non-Index Cancer Death		Non-Cancer Death		Censor
	Incidence	Death	5-Year Crude Observed Survival Rate	Death	5-Year Corrected Survival Rate	Death	5-Year Corrected Survival Rate	Death	5-Year Corrected Survival Rate	
	*n*	*n*	% (95% CI)	*n*	% (95% CI)	*n*	% (95% CI)	*n*	% (95% CI)	*n*
All	220,859	107,061	51.53 (51.32–51.74)	86,938	–	11,483	–	8640	–	113,798
Mouth, pharynx	3336	1455	56.33 (54.63–58.00)	1107	65.22 (63.52–66.85)	180	92.87 (91.78–93.82)	168	93.03 (91.92–93.99)	1881
Esophagus	2517	1742	30.69 (28.89–32.50)	1543	36.03 (34.09–37.96)	66	94.59 (93.07–95.79)	133	90.08 (88.23–91.66)	775
Stomach	28,526	15,311	46.31 (45.73–46.89)	13,286	51.98 (51.39–52.57)	600	96.62 (96.33–96.88)	1425	92.23 (91.83–92.62)	13,215
Colorectum	33,782	15,734	53.47 (52.93–54.00)	13,602	58.50 (57.96–59.03)	673	97.18 (96.96–97.39)	1459	94.06 (93.75–94.36)	18,048
Liver	13,923	10,689	23.24 (22.54–23.95)	8970	29.70 (28.89–30.52)	731	91.38 (90.70–92.02)	988	85.69 (84.78–86.56)	3234
Gallbladder	6531	5713	12.48 (11.69–13.29)	5228	15.39 (14.48–16.33)	271	89.43 (87.90–90.77)	214	90.74 (89.17–92.09)	818
Pancreas	8372	7917	5.31 (4.84–5.80)	7536	6.22 (5.69–6.78)	210	91.65 (89.85–93.14)	171	93.14 (91.43–94.52)	455
Larynx	269	87	67.63 (61.68–72.86)	50	80.37 (74.93–84.76)	21	90.73 (86.10–93.87)	16	92.75 (88.39–95.52)	182
Lung/bronchial	20,470	14,821	27.56 (26.95–28.18)	13,712	30.59 (29.94–31.24)	548	95.20 (94.75–95.60)	561	94.68 (94.20–95.12)	5649
Bone, joints, articular cartilage	992	415	58.22 (55.08–61.22)	301	67.48 (64.34–70.40)	93	88.78 (86.38–90.78)	21	97.20 (95.71–98.17)	577
Skin	2781	792	71.48 (69.76–73.12)	259	90.02 (88.80–91.12)	157	93.48 (92.42–94.41)	376	84.94 (83.47–86.30)	1989
Breast	45,842	7958	82.63 (82.28–82.97)	6265	86.08 (85.76–86.40)	537	98.70 (98.58–98.80)	1156	97.26 (97.09–97.41)	37,884
Cervix uteri	8292	2796	66.16 (65.12–67.16)	2147	72.97 (71.98–73.94)	420	93.89 (93.29–94.43)	229	96.57 (96.10–96.98)	5496
Corpus uteri	6577	1772	73.04 (71.95–74.10)	1151	81.67 (80.69–82.61)	483	91.73 (90.99–92.41)	138	97.50 (97.05–97.88)	4805
Ovary	6721	3697	45.01 (43.82–46.20)	3362	48.43 (47.20–49.64)	236	95.07 (94.40–95.67)	99	97.78 (97.29–98.18)	3024
Kidney, urinary tract	3574	1680	52.95 (51.30–54.57)	1214	63.84 (62.18–65.45)	282	89.70 (88.47–90.79)	184	92.50 (91.36–93.49)	1894
Bladder	2819	1342	52.40 (50.54–54.23)	989	62.95 (61.07–64.76)	167	91.82 (90.52–92.94)	186	90.69 (89.29–91.91)	1477
Brain, central nervous system	1700	1208	28.82 (26.68–30.99)	579	53.50 (50.58–56.33)	501	61.61 (58.75–64.34)	128	87.54 (85.12–89.59)	492
Thyroid	5071	676	86.66 (85.70–87.57)	471	90.59 (89.75–91.37)	86	98.17 (97.74–98.51)	119	97.45 (96.96–97.87)	4395
Malignant lymphomas, Hodgkin lymphomas	6259	2901	53.67 (52.43–54.90)	2445	59.65 (58.40–60.88)	211	95.34 (94.67–95.93)	245	94.39 (93.65–95.05)	3358
Multiple myeloma	1495	1038	30.49 (28.17–32.84)	869	36.84 (34.26–39.43)	79	91.84 (89.78–93.50)	90	90.14 (87.85–92.02)	457
Leukemias	3215	2012	37.61 (35.93–39.29)	1850	40.94 (39.19–42.68)	64	96.88 (95.98–97.59)	98	94.85 (93.69–95.80)	1203

Excluded other cancer *n* = 7795, 95% CI denotes 95% confidence intervals. All survival rates in the table are crude survival rates.

## Data Availability

The data that support the findings of this study are not publicly available due to privacy and ethical restrictions. The data are available from the corresponding author on reasonable request.

## Appendix A. Cause of Death Classification (Code) As Defined by the Ministry of Health, Labour and Welfare

**Table A1:** Cause of death classification (code).

Cause of Death Classification (Code) As Defined by the Ministry of Health, Labour and Welfare		
Certain infectious and parasitic diseases (1000)	Prostate (2115)	Intracerebral hemorrhage (9302)
Lip, oral cavity and pharynx (2101)	Bladder (2116)	Cerebral infarction (9303)
Esophagus (2102)	Central nervous system (2117)	Other cerebrovascular diseases (9304)
Stomach (2103)	Malignant lymphoma (2118)	Aortic aneurysm and dissection (9400)
Colon (2104)	Leukaemia (2119)	Other diseases of the circulatory system (9500)
Rectosigmoid junction and rectum (2105)	Other malignant neoplasms, stated or presumed to be primary, of lymphoid, hematopoietic and related tissue (2120)	Diseases of the respiratory system (10,000)
Liver and intrahepatic bile ducts (2106)	Other malignant neoplasms (2121)	Diseases of the digestive system (11,000)
Gallbladder and other biliary tracts (2107)	In situ neoplasms and benign neoplasms and neoplasms of uncertain or unknown behavior (2200)	Diseases of the genitourinary system (14,000)
Pancreas (2108)	Diseases of the blood and blood-forming organs and certain disorders involving the immune mechanism (3000)	Accidents (20,100)
Larynx (2109)	Endocrine, nutritional and metabolic diseases (4000)	Suicide (20,200)
Trachea, bronchus and lung (2110)	Mental and behavioral disorders (5000)	Homicide (20,300)
Skin (2111)	Diseases of the nervous system (6000)	Other external causes (20,400)
Breast (2112)	Hypertensive diseases (9100)	Other (23,000)
Uterus (2113)	Heart diseases (excluding hypertensive heart diseases) (9200)	
Ovary (2114)	Subarachnoid hemorrhage (9301)	

## Appendix B. Relationships between Cancer Sites and Cause of Death by Sex

The diagram above illustrates the correlation between the site of cancer incidence and the specific cause of death. The left side of the diagram represents the site of cancer incidence, while the right side represents the classification of causes of death. The number before the cause of death is the code for the cause of death classification as defined by the Japanese Ministry of Health, Labour and Welfare. The height (width of the bands) represents the number of individuals, with thicker bands indicating higher numbers. It is evident that deaths due to index cancer are prevalent, but for causes other than cancer, heart disease and respiratory system diseases are more common.
